# Factors Influencing Vitamin D Levels in Neonatal Umbilical Cord Blood: A Two-Center Study From Tibet and Shenyang

**DOI:** 10.3389/fped.2020.543719

**Published:** 2020-11-23

**Authors:** Mingli Yu, Xiuxiu Liu, Jiujun Li

**Affiliations:** ^1^Department of Pediatrics, Shengjing Hospital of China Medical University, Shenyang, China; ^2^Department of Pediatrics, Naqu People's Hospital, Naqu, China; ^3^Plateau Medical Research Center of China Medical University, Department of Pediatrics, Shengjing Hospital of China Medical University, Shenyang, China

**Keywords:** vitD deficiency, neonates, mothers, vitD supplementation, high altitude

## Abstract

**Objective:** To investigate the factors influencing the levels of vitamin D (vitD) in the umbilical cord blood of neonates born in Naqu, Tibet (4,500 m above sea level), and Shenyang, Liaoning Province (500 m above sea level).

**Methods:** This prospective study was conducted from June 2017 to October 2018 in Naqu (the plateau group) and Shenyang, (the non-plateau group). Healthy mothers that gave birth to healthy neonates of >2,000g after 38 weeks' gestation were enrolled in the study, as were their neonates. After separation of serum from the umbilical cord and mothers for routine biochemical tests, discarded samples were remained for analyses of vitD, calcium, phosphorus, alkaline phosphatase (ALP) and parathyroid hormone (PTH). Questionnaires were developed covering the demographic characteristics and possible risk factors for neonatal vitD deficiency of mothers. Statistical analysis was performed to identify associations between the calcium, phosphorus, ALP, PTH, maternal factors and neonatal vitD levels.

**Results:** In total, 295 neonates and 225 mothers were enrolled in the study. VitD deficiency was common in neonates and mothers. The risk of vitD deficiency was higher in the plateau group than in the non-plateau group. The mean levels of 25-hydroxy vitD (25(OH)D) in mothers and neonates from the plateau group were 8.49 ± 4.12 ng/mL and 10.17 ± 5.07 ng/mL, respectively. Such levels were significantly lower than those in the non-plateau group (19.77 ± 9.57 ng/mL and 23.93 ± 11.01 ng/mL, respectively). The vitD levels of neonates and mothers were highest in the summer and lowest in the winter. Cord blood vitD was positively correlated with the vitD levels in mothers' serum (*r* = 0.75, *P* < 0.05). Increased PTH levels in mothers and decreased cord blood calcium levels were risk factors for neonatal vitD deficiency. A lack of vitD supplementation during pregnancy was associated with an 8.91-fold higher probability of neonatal vitD deficiency (OR = 8.91, 95% CI = 1.521–9.429, *P* < 0.001).

**Conclusions:** The levels of neonatal and maternal vitD in the plateau group were generally lower than those in the non-plateau group. VitD supplementation during pregnancy could effectively reduce the risk of vitD deficiency in neonates.

## Introduction

VitD is not only an important steroid hormone, but also a lipid-soluble vitamin, which has a wide range of biological effects, including regulatory effects on embryonic organ development, cell proliferation, differentiation, and maturation (1). A lack of vitD during pregnancy is the most important risk factor for infantile rickets, and may also result in poor fetal growth and neonatal development (2). Although the VitD supplementation to pregnant women is encouraged by numerous published guidelines for its prevention (3, 4), sunlight-produced VitD in the skin has played a critically important role (5) and the altitude has a dramatic influence on previtamin D_3_ synthesis as well (6). When an adult wearing a bathing suit is exposed to one minimal erythemal dose of ultroviole radiation (a slight pinkness to the skin 24 h after exposure), the amount of vitamin D produced is equivalent to ingesting between 10,000 and 25,000 IU (7). Low exposure to sun, atmospheric pollution, low physical activity, indoor confinement during the day and high buildings are common risk factors of VitD deficiency in the pregnant women (8). These risk factors are infrequent at high altitudes regions as compared to plain areas. Nevertheless, VitD deficiency occurs mostly in higher altitudes (9). The prevalence of vitD deficiency during pregnancy and in neonates at high altitude (1,900–2,200 m above sea level) is higher than those in plains (10). Among nomads in Tibet (4,500 m above sea level), the 25(OH)D status is alarmingly low (11). Besides, the pregnancy alone increases the risk of vitamin D deficiency (8, 12).

There is no investigation on the vitD status among pregnant women and neonates from Tibet, the ultrahigh-altitude region. Nor have there been large-scale national survey data to report the vitD status of neonates in China. This study investigated the demographic characteristics and possible risk factors that may affect the levels of vitD, as well as tested the levels of 25(OH)D, calcium, phosphorus, ALP, and PTH in umbilical cord blood and pregnant women living in ultrahigh-altitude regions (Naqu, Tibet, 4,500 m above sea level) and the plain (Shenyang, Liaoning, 500 m above sea level). Using such data, our study aimed to fill the knowledge gap of maternal and neonatal vitD status in the ultrahigh-altitude region, as well as explore the correlation of maternal-neonatal pairs with vitD levels. Moreover, the results from regional studies are critical to develop appropriate prevention strategies during pregnancy, to optimize vitamin D status of mothers and neonates, in a region-specific manner.

## Methods and Materials

### Subjects

From June 2017 to October 2018, our study recruited 225 mothers and 295 neonates delivered at the Naqu People's Hospital, Tibet (the plateau group) and the Shengjing Hospital of China Medical University, Shenyang (the non-plateau group). Healthy mothers who gave birth to healthy neonates weighing >2,000 g after 38 weeks' gestation were included, as were their neonates. Neonates with genetic metabolic diseases, infections, asphyxia, anemia, and intrauterine growth restriction, or those who were born premature were excluded from the study. Mothers with an infection during pregnancy and those who had an early membrane rupture, chorioamnionitis, preeclampsia, hypertension, gestational diabetes, chronic diarrhea, liver diseases, kidney diseases, parathyroid diseases, and other calcium-modifying conditions were also excluded. The study was approved by the Ethics Committee of the Shengjing Hospital of China Medical University (Protocol Identification Number: 2017PS33K).

### Sample Collection

When the pregnant women are hospitalized for labor (within 3 days before delivery), 2 mL of venous blood for biochemical tests is taken routinely. It is difficult to collect blood from neonates, thus the samples for blood gas ion analysis and biochemical tests for neonates are from umbilical cord immediately following birth. After the separation of serum for biochemical tests, the remaining serum is retained in the test center. The samples used in our study were all discarded samples after the clinical routine diagnosis and treatment, which had no impact on the routine diagnosis and treatment of patients, and stored at −80°C until analysis at the Clinical Test Center of Shengjing Hospital. Samples from Naqu were sent to the same laboratory by the Cold-chain transportation. The gestational age, birth weight, body length, head circumference, and chest circumference of neonates were measured and recorded, as was the age, gestational times, delivery times, and the details of vitD supplementation for mothers.

Serum 25(OH)D levels were analyzed by an electrochemiluminescence immunoassay (competitive inhibition method) using the Roche E602 device. Serum PTH was also analyzed by electrochemiluminescence immunoassays (double antibody sandwich method) using the Roche E602 device. Serum calcium was detected using the methyl dimethyl phenol blue (MXB) method, phosphorus was detected using the molybdate method (UV absorption method of phosphomolybdic acid), and ALP was detected using the *P*-nitrobenzene method of phosphoric acid using with the Abbott I16200 device. Adult reference ranges are calcium (1.9–2.6 mmol/L), phosphate (1.2–1.9 mmol/L), ALP (40–375 U/L), and PTH (15–65 pg/mL).

### Identification of 25(OH)D Levels

A cord blood serum 25(OH)D level <12 ng/mL (30 nmol/L) was considered as vitD deficiency, 12–20 ng/mL (30–50 nmol/L) was considered as vitD insufficiency, and >20 ng/mL (50 nmol/L) was considered as vitD sufficiency (13). In pregnant women, vitD deficiency was defined as a 25(OH)D level below 20 ng/mL (50 nmol/L), while vitD insufficiency was defined as a 25(OH)D level of 21–29 ng/mL (52.5–72.5 nmol/L) (4).

### Statistical Analysis

SPSS 23.0 (SPSS, Chicago, IL, USA) was used for the processing and statistical analysis of the data. Numeric data were shown as the mean and standard deviation. Categorical data were summarized as number and percentage. The Mann-Whitney *U* test and Kruskal-Wallis test were used for statistical analyses of non-parametric data. Parametric variables were analyzed using Student's *t*-tests and one-way analysis of variance. Unitary linear regression analysis was used to determine the association between each potential risk factor and serum 25(OH)D levels. Binary logistic regression analysis was used to calculate the odds ratios (OR) concerning vitD deficiency. A *P*-value of 0.05 was considered statistically significant.

## Results

### General Information

There were 295 umbilical cord blood samples collected and 225 were paired with maternal samples. A total of 47 neonates (26 males; 21 females) and 47 mothers were included from the Naqu People's Hospital of Tibet, which formed the plateau group, and 248 neonates (121 males; 127 females) and 178 mothers were included from the Shengjing Hospital of China Medical University, Shenyang, Liaoning Province, which formed the non-plateau group. Among the 295 neonates, the average gestational age was 39.70 ± 0.83 weeks (range, 38–41.29 weeks), and the average birth weight was 3,319.85 ± 397.36 g (range, 2,100–4,475 g). Maternal and neonatal characteristics according to either regional group are shown in Table 1. There was no significant difference in the baseline characteristics between the plateau and non-plateau groups.

**Table 1:** Baseline characteristics between the plateau and non-plateau groups.

	**Plateau**	**Non-plateau**	***t/*χ^2^-value**	***P***
Neonatal BMI (kg/m^2^)	12.97 ± 1.74	13.07 ± 1.53	−6.811	0.093
gestational age (week)	39.64 ± 0.87	39.78 ± 0.82	−0.628	0.531
Gender (male/female)	26/21	121/127	0.674	0.412
Medical insurance (yes/no)	45/2	238/10	0.005	0.943
Mothers age	28.85 ± 5.06	29.34 ± 4.23	−3.222	0.101
Gestational times	3.03 ± 1.48	1.72 ± 0.84	9.692	0.245
Delivery times	2.58 ± 1.12	1.57 ± 0.59	15.677	0.216

*BMI for Body Mass Index*.

**Figure 1:**
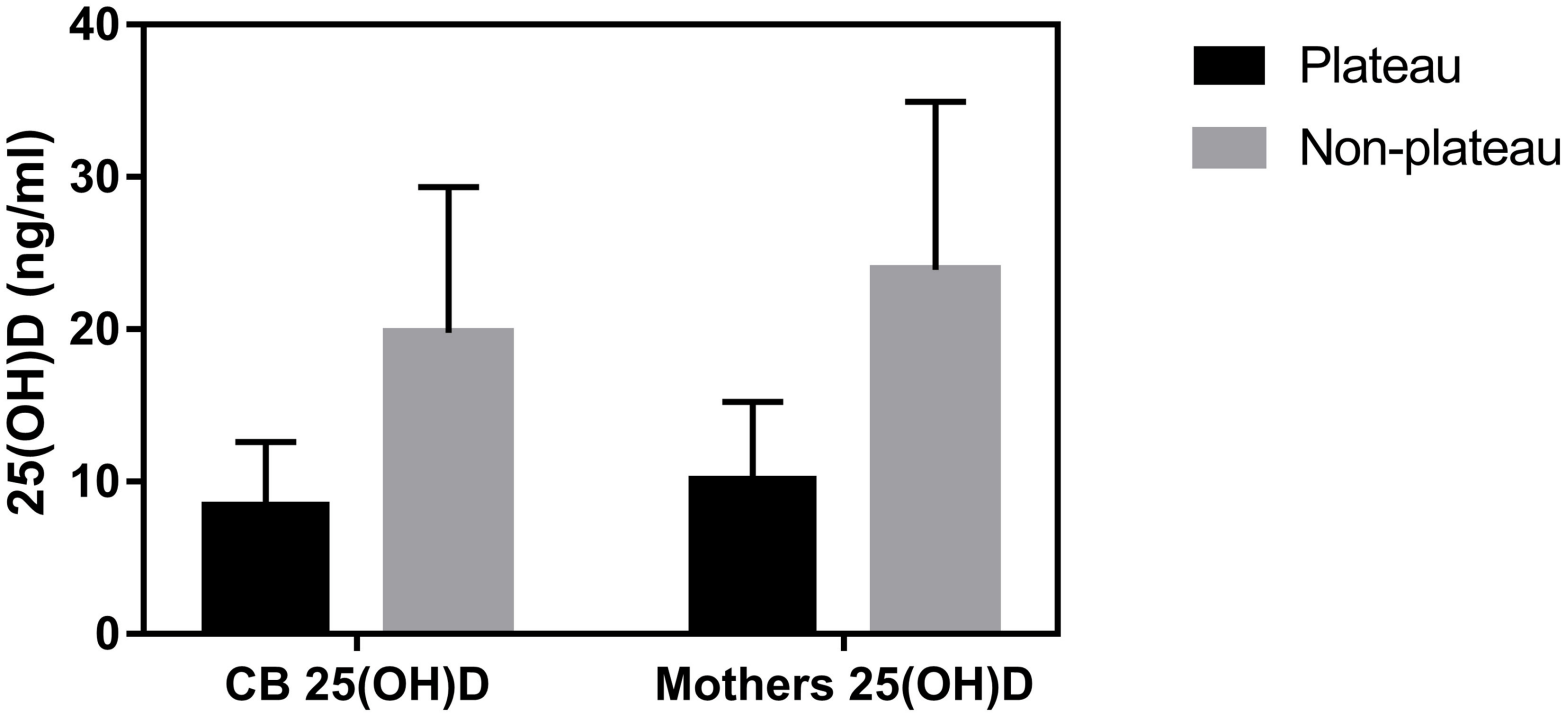
The average 25(OH)D levels of cord blood and mothers between the plateau and non-plateau groups.

Among the 47 neonates in the plateau group, 39 (82.98%) exhibited 25(OH)D levels below 12 ng/mL, while seven (14.89%) exhibited 25(OH)D levels between 12–20 ng/mL, and only one (2.13%) exhibited a level above 20 ng/mL. Among the 248 neonates from the non-plateau group, 51 (20.56%) exhibited 25(OH)D levels below 12 ng/mL, while 91 (36.69%) exhibited 25(OH)D levels between 12–20 ng/mL, and 106 (42.75%) had 25(OH)D levels above 20 ng/mL. The average cord blood 25(OH)D level in the plateau group was 8.49 ± 4.12 ng/mL, which was significantly lower than that in the non-plateau group (19.77 ± 9.57 ng/mL). A statistically significant difference was observed in vitD levels from neonatal cord blood samples between the plateau and the non-plateau groups (*P* < 0.05). Among the 47 mothers in the plateau group, 44 (93.62%) exhibited 25(OH)D levels below 12 ng/mL, while three (6.38%) exhibited 25(OH)D levels between 12–20 ng/mL, and none had levels above 20 ng/mL. The average 25(OH)D level was 10.17 ± 5.07 ng/mL. As for the 178 mothers in the non-plateau group, 24 (13.48%) exhibited 25(OH)D levels below 12 ng/mL, while 53 (29.78%) exhibited 25(OH)D levels between 12–20 ng/mL, and 101 (56.74%) had 25(OH)D levels above 20 ng/mL. The average 25(OH)D level of mothers in the non-plateau group was 23.93 ± 11.01 ng/mL, which was significantly higher than that in the plateau group (*P* < 0.05; Figure 1, Table 2).

**Table 2:** Vitamin D status of cord blood and mothers between the plateau and non-plateau groups.

	**Plateau**	**Non-plateau**
CB 25(OH)D (≤12 ng/mL) (deficient)	39 (82.90%)	51 (20.56%)
CB 25(OH)D (12–20 ng/mL) (insufficient)	7 (14.90%)	91 (36.69%)
CB 25(OH)D (≥20 ng/ml) (normal level)	1 (2.1%)	106 (42.75%)
Mothers 25(OH)D (≤20 ng/ml) (deficient)	44 (93.62%)	24 (13.48%)
Mothers 25(OH)D (20–29 ng/ml) (insufficient)	3 (6.38%)	53 (29.78%)
Mothers 25(OH)D (30–100 ng/ml) (normal level)	0	101 (56.74%)

*CB for cord blood*.

When groups were classified based on cord blood 25(OH)D levels by 20 ng/mL, categorized as deficient (<20 ng/mL) and normal (≥20 ng/mL), there were statistically significant differences in the levels of cord blood calcium, phosphorus, ALP, and PTH between the two groups, as well as in the age, gestational times, delivery times, and PTH and vitD levels among mothers (*P* < 0.05; Table 3). In three clinically normal neonates with very low 25(OH)D status (4.60, 4.83, and 5.18 ng/mL, respectively), their calcium, phosphate and ALP were in normal range. In one (1/295) clinically normal neonate with low 25(OH)D, 10.89 ng/mL, his calcium, phosphate and ALP (161.5U/L) were in normal range, except high PTH (91.3 pg/mL). 98.98% of the neonates had PTH lower than the normal adult range (15–65 pg/mL). However, none of the neonates had hypocalcemia. The mean values of serum calcium, phosphate, ALP, and PTH levels in mothers were within normal range. Neonates within 25(OH)D levels <20 ng/mL had higher ALP and PTH levels compared with the normal 25(OH)D group, so as their mothers.

**Table 3:** Univariate analysis of independent factors in neonatal cord blood vitamin D.

**Variable**	**CB Vitamin D deficiency group (*N* = 188)**	**CB Normal vitamin D group (*N* = 107)**	***t***	***p***
Birth weight (g)	3318.5 ± 4375.9	3322.2 ± 3164.1	−0.076	0.067
CB PTH (pg/ml)	5.16 ± 2.06	4.27 ± 2.60	−2.762	0.007
CB phosphorus (mmol/L)	1.81 ± 0.69	1.68 ± 0.29	1.823	0.028
CB calcium (mmol/L)	2.25 ± 0.42	2.35 ± 0.23	−2.215	0.001
CB ALP (U/L)	158.88 ± 70.67	146.04 ± 44.41	1.698	0.001
Mothers age	29.34 ± 4.26	29.85 ± 3.29	−1.081	0.010
Gestational times	1.90 ± 0.67	1.54 ± 0.62	2.644	<0.001
Delivery times	1.64 ± 0.51	1.18 ± 0.45	3.837	<0.001
Mothers 25(OH)D (ng/mL)	15.30 ± 7.03	29.86 ± 12.46	−10.666	<0.001
Mothers PTH (pg/mL)	23.26 ± 10.85	18.11 ± 8.37	3.339	0.001
Mothers ALP (U/L)	191.14 ± 81.67	162.81 ± 87.50	2.757	0.559
Mothers phosphorus (mmol/L)	1.20 ± 0.34	1.10 ± 0.33	2.325	0.549
Mothers calcium (mmol/L)	2.19 ± 0.14	2.19 ± 0.21	−0.035	0.136

*CB for cord blood; PTH for parathyroid hormone; ALP for alkaline phosphatase*.

### Calcium, Phosphorus, ALP, and PTH Levels of Cord Blood in Naqu (the Plateau Group) and Shenyang (the Non-plateau Group), and Their Correlations

The levels of umbilical cord blood phosphorus and ALP were higher in the plateau group than in the non-plateau group, while birth weight was lower in the plateau group than in the non-plateau group. The differences were statistically significant (*P* < 0.05). Cord blood calcium levels were higher in the plateau group than those in the non-plateau group, while PTH levels were lower in the plateau group than in the non-plateau group. However, both differences were not statistically significant (*P* > 0.05; Table 4).

**Table 4:** 25(OH)D, calcium, phosphorus, ALP, PTH of cord blood and neonatal birth weight between the plateau and non-plateau groups.

	**Calcium (mmol/L)**	**Phosphorus (mmol/L)**	**ALP (U/L)**	**PTH (pg/mL)**	**Birth weight (g)**
Plateau (*N* = 47)	2.34 ± 0.20	2.05 ± 0.53	212.04 ± 69.78	1.56 ± 0.95	2988.98 ± 363.75
Non-plateau (*N* = 248)	2.28 ± 0.39	1.71 ± 0.57	143.27 ± 54.86	4.82 ± 2.36	3371.34 ± 426.78
*t*	1.111	3.761	7.523	−4.732	−5.847
*p*	0.267	<0.01	<0.01	0.091	<0.01

*PTH for parathyroid hormone; ALP for alkaline phosphatase*.

### Correlations Between Components of the Calcium Metabolic System and VitD in Neonates

There was no linear correlation between cord blood vitD levels and cord blood PTH, phosphorus, and ALP levels (*P* > 0.05). Cord blood calcium levels were positively correlated with cord blood vitD levels, but the correlation coefficient was small (*R* = 0.185, *P* < 0.01). No linear correlation was observed between cord blood PTH and cord blood calcium, phosphorus, and ALP levels (*P* > 0.05; Table 5).

**Table 5:** Correlations between components of the calcium metabolic system and vitamin D in cord blood.

		**25(OH)D**	**Calcium**	**Phosphorus**	**ALP**
PTH	R	−0.003	0.106	−0.176	0.045
	*P*	0.964	0.069	0.052	0.446
Calcium	R	0.185			
	*P*	0.001			
Phosphorus	R	−0.096			
	*P*	0.099			
ALP	R	−0.092			
	*P*	0.114			

*PTH for parathyroid hormone; ALP for alkaline phosphatase*.

### Correlation of VitD Metabolic System Components Between Neonates and Mothers

Cord blood vitD level was positively correlated with mothers' serum vitD level (*r* = 0.75, *P* < 0.05) and inversely weakly correlated with mothers' gestational times, and delivery times (*r* = 0.175, 0.278, respectively, *P* < 0.05 for both). The levels of calcium and phosphorus in cord blood were positively weakly correlated with mothers' serum vitD level (*P* < 0.05; Table 6). Cord blood vitD level was positively correlated with the levels of mothers' calcium, phosphorus, but inversely correlated with the levels of mothers' ALP and PTH, both weakly, (*P* < 0.05; Table 6).

**Table 6:** Correlation of vitamin D metabolic system components between mothers and cord blood.

		**Mothers 25(OH)D**	**Mothers calcium**	**Mothers phosphorus**	**Mothers ALP**	**Mothers PTH**
CB 25(OH)D	R	0.75	0.134	0.141	0.127	0.221
	*P*	<0.001	0.044	0.034	0.032	0.001

*CB for cord blood; PTH for parathyroid hormone; ALP for alkaline phosphatase*.

### VitD Levels of Neonates and Mothers in Different Seasons

The blood collection time was divided into four seasons, including spring (March to May); summer (June to August); autumn (September to November); and winter (December to February). A significant seasonal difference in vitD levels was observed between cord blood and mothers' serum (*F* = 3.446 and 6.890, respectively, *P* < 0.05 for both). VitD levels in cord blood and mothers' serum were highest in the summer, with mean concentrations of 22.12 ± 7.97 ng/mL and 28.16 ± 10.47 ng/mL, respectively. Additionally, vitD levels in cord blood and mothers' serum were lowest in the winter, with mean concentrations of 17.70 ± 6.50 ng/mL and 19.47 ± 4.59 ng/mL, respectively (Table 7).

**Table 7:** Vitamin D levels of neonates and mothers in different seasons.

**Seasons**	**Neonates number**	**CB 25(OH)D**	**F(*P*) value**	**Mothers number**	**Mothers 25(OH)D**	**F(*P*) value**
Spring	21	19.00 ± 7.37	F = 3.446	21	24.44 ± 9.21	F = 6.890
Summer	87	22.12 ± 7.97	*P* = 0.018	66	28.16 ± 10.47	*P* < 0.01
Autumn	84	19.29 ± 6.73		55	21.28 ± 10.07	
Winter	21	17.70 ± 6.50		19	19.47 ± 4.59	

### The Correlations of Independent Factors With 25(OH)D Levels by Multivariate Analysis

There was no multicollinearity among neonatal birth weight, cord blood PTH, calcium, phosphorus, and ALP, or maternal serum vitD, PTH, calcium, phosphorus and ALP levels. There was no significant difference in the effects of maternal age, gestational times, delivery times, or PTH, ALP and phosphorus levels on cord blood vitD levels by logistic regression analysis (*P* > 0.05). Decreased cord blood calcium levels and increased PTH levels in mothers were risk factors for neonatal vitD deficiency. Mothers without vitD supplementation during pregnancy were associated with an 8.91-fold higher probability of neonatal vitD deficiency at birth (OR = 8.91, 95% CI = 1.521–9.429, *P* < 0.001). The risk of neonatal vitD deficiency was 14.11-fold higher for neonates born in the plateau area, compared to neonates born in the non-plateau area (OR = 14.11, 95% CI = 1.055–7.571, *P* = 0.015).

Decreased cord blood calcium (by 1 mmol/L) was associated with a 4.69 (1/0.213)-fold higher probability of neonatal vitD deficiency at birth (OR = 0.213, 95% CI = 0.069–0.660, *P* = 0.007). The risk of cord blood vitD deficiency decreased by 13.5% for every unit of maternal vitD increase (OR = 0.881, 95% CI = 0.845–0.918, *P* < 0.001), but increased by 5.9% for every unit of maternal PTH increase (OR = 1.059, 95% CI = 1.015–1.105, *P* = 0.008; Table 8).

**Table 8:** Multivariate analysis for factors associated with vitamin D deficiency in cord blood.

**Variable**	**B**	**Waldχ^2^**	***P***	**OR**	**95% CI**
Born in plateau	2.647	5.864	0.015	14.11	1.055–7.571
Without VitD supplementation during pregnancy	2.187	7.086	0.008	8.91	1.521–9.429
Mothers 25(OH)D	−0.127	36.355	<0.001	0.881	0.845–0.918
Mothers PTH	0.058	7.142	0.008	1.059	1.015–1.105
Mothers age	0.005	0.007	0.935	1.005	0.899–1.122
Gestational times	0.045	0.042	0.838	1.046	0.679–1.611
Delivery times	0.191	0.196	0.658	1.210	0.520–2.815
CB calcium	−1.545	7.189	0.007	0.213	0.069–0.660
CB phosphorus	1.109	3.127	0.077	3.032	0.887–10.370
CB ALP	−0.001	0.096	0.756	0.999	0.994–1.005
CB PTH	−0.077	1.172	0.279	0.926	0.806–1.064

*CB for cord blood, PTH for parathyroid hormone; ALP for alkaline phosphatase*.

## Discussion

This is the first survey of vitD levels in the umbilical cord blood of neonates and their mothers in the ultrahigh altitude (4,500 m above sea level) area of Naqu, Tibet, as well as a large-scale national survey data to report the maternal and neonatal vitD status in China. Although the prevalence of vitD deficiency in mothers and neonates has been widely reported throughout the world, studies on the components of the vitD metabolism system (calcium, phosphorus, ALP, PTH) between mothers and neonates are rare. Our study included 47 pairs of mother-neonate samples from the Naqu plateau and 178 pairs of mother-neonate samples from the Shenyang non-plateau area. The level of cord blood 25(OH)D in Naqu (8.49 ± 4.12 ng/mL) was very low. It has been reported in other regions, the values of cord blood 25(OH)D were 31.0 ± 12.5 nmol/L in Zhejiang (14), 29.77 ± 12.51 nmol/L in Shanghai (15), 31.58 ± 12.72 ng/mL in Guangzhou (16) and 14.4 ± 6.7 ng/mL in Beijing (17). Vitamin D deficiency is also common in neonates and their mothers in Europe (18) and America (19). In our study, nearly half of mothers and their neonates in the non-plateau group exhibited vitD deficiency, by contrast, that prevalence increased to 100% in mothers and 97.88% in neonates residing in the plateau. The risk of vitD deficiency in neonates born in Naqu (plateau) was significantly higher than that for neonates born in Shenyang (non-plateau area). Based on previous findings (10), the geographical location at northern hemisphere and high altitude (1,900–2,200 m above sea level), might have contributed to the high prevalence of neonatal and maternal hypovitaminosis D during pregnancy. It was also found *in vitro* ampoule experiments at Everest, the production of previtamin D_3_ was associated with altitude (6). Naqu is located at northern hemisphere and extremely high altitude, where the intensity of ultraviolet radiation is greater than that of the Shenyang non-plateau area. However, the annual average temperature is −2.2°C in Naqu, and the need for heavy traditional Tibetan clothing limits the body surface exposed to ultraviolet B radiation (UVB). Compared to pregnant women in the non-plateau area, the skin color of mothers in Naqu is darker, which is adverse for vitamin D synthesis. The vitD content in a traditional Tibetan diet is rare, including highland barley, wheat, beef, mutton, and ghee (11). Thus, all such factors have been associated with an increased prevalence of neonatal and maternal hypovitaminosis D during pregnancy (20, 21).

Our study found that neonatal vitD had a positive correlation with maternal vitD, so did neonatal calcium and phosphorus, which was consistent with cardinal features in fetal vitD metabolic system (22, 23). For neonates, maternal-fetal transport is the main source of vitD, and maternal vitD reserves during pregnancy determine the level of vitD in cord blood. Maternal hypovitaminosis D significantly increased the risk of neonatal vitD deficiency (14, 15, 18) and poor development of neonatal bone mineralization at birth, also observed in this study. The 25(OH)D concentrations in cord blood were 60–70% of their mothers' values, but optimal cord blood 25 (OH)D is not known (23, 24).

Hypovitaminosis D in many neonates may not be clinically relevant unless there is associated biochemical evidence of vitamin D deficiency (25). In this study, there was also a tendency to higher serum ALP and PTH levels in neonates with hypovitaminosis D than the values in normal-25(OH)D group. It is similar to findings in early or asymptomatic cases of vitamin D deficiency rickets (25, 26). Bone metabolic activity as measured by ALP levels was occasionally increased in patients with low 25(OH)D levels, consistent with previous studies (27–29). VitD plays an important role in the process of fetal growth, and cooperates with PTH to maintain calcium homeostasis. Long-term vitD deficiency can lead to increased PTH concentrations (30, 31). PTH is expressed in the placenta, regulates the placental expression of genes involved in the transfer of calcium and other solutes, and may directly stimulate placental calcium transfer (32). Despite many adaptations in the vitD-calcium metabolic system during pregnancy, the inverse relationship between 25(OH)D and PTH is retained, or only slightly weakened (33). In our study, neonates with hypovitaminosis D had an increase of PTH concentrations—though lower than the normal adult range, which might result from an intrauterine adaptive up-regulation of fetal PTH, as a result of maternal-fetal hypovitaminosis D, to maintain adequate calcium supply for the fetus development (34, 35). Besides, it has been previously reported that there was no correlation between 25 (OH)D and PTH in cord blood (36, 37), indicating that the well documented increase of PTH in adults with secondary hyperparathyroidism, is not evident in neonates after birth, due to temporary suppression of PTH (35). Thus, PTH levels in cord blood could be lower than the normal adult range as long as no hypocalcemia happened (35, 38), in the condition of suppression by active placental calcium transport (39). Increased PTH levels in mothers and decreased cord blood calcium levels could indicate neonatal risk of vitD deficiency. Studies on the threshold of ALP and PTH under subclinical hypovitaminosis D are limited and variable (40). Further studies are necessary to evaluate the magnitude of neonatal ALP and PTH variation under circumstances of vitamin D status at birth.

Our results demonstrated a seasonal variation in 25(OH)D. The vitD levels in cord blood and mothers were highest in summer and lowest in winter. Such findings may arise from increases in body surface exposure to sun during the summer. Seasons at sampling (autumn/winter) were reported as factors related to deficient 25(OH)D concentrations (41). Decreases in time spent outdoors, skin pigmentation, coverage and aging can all lead to insufficient vitD production (42–44). Increasing casual sun exposure for reaching the optimal serum 25(OH)D levels has been recommended (45). However, as excessive UV radiation is a carcinogen, it might be worth obtaining additional vitamin D from foods or supplements (3).

Our finding confirms that the main determinant of the vitamin D level in a neonate is the vitamin D level of the mother (46). The risk of neonatal vitD deficiency was increased almost nine-times in mothers who did not take vitD supplements during pregnancy. Strategies should be developed to prevent maternal and neonatal vitamin D deficiency. Daily prenatal vitamin D supplementation should be promoted, particularly in the 3rd trimester when the majority of placental vitamin D transfer to the fetus occurs, with the women who have insufficient sunlight exposure taken into account (39). The current recommended intake for vitamin D during pregnancy is at least 600 IU/day of vitamin D by both the Endocrine Society (4) and the Institute of Medicine (47). And Hollis's study showed that vitamin D supplementation of 4,000 IU/day for pregnant women was safe and most effective (12). With the addition of calcium supplement in pregnancy, mothers had higher maternal concentrations of vitD (48), which had a positive effect on neonatal vitD levels (49). Supplementation of 400 IU/day (10 μg) of vitD is adequate to prevent rickets and recommended for all infants from birth to 12 months of age, independent of their mode of feeding (13).

This study had several limitations. First, maternal data that may correlate with neonatal vitD levels at birth, such as the pre-pregnancy body mass index, skin color, and sun exposure during pregnancy were not collected. However, such limitations were unlikely to affect the vitD levels of neonates at birth (50). Secondly, our analyses lacked a detailed evaluation of dietary protein and fat intake as potential food sources of vitD. Finally, the sample size from Naqu was relatively small, compared to that from Shenyang and other related studies, which may have biased the statistical analysis.

## Conclusions

In conclusion, this study reported results from a prospective maternal-neonatal cohort study between the Naqu plateau and Shenyang non-plateau area, that mainly focused on neonatal vitD deficiency. The study demonstrated a high prevalence of vitD deficiency in mothers and neonates in both populations, which was purely derived from the >95% of individuals from Naqu and 50% from Shenyang who had vitD deficiency/insufficiency, and revealed the particularly higher risk of such deficiency in individuals from Naqu, Tibet. VitD supplementation should be provided to pregnant women with risk factors based on their place of residence and lifestyle, such as plateau areas. Our study also provided data on the specific-population characteristics for developing recommendations to prevent neonatal vitD deficiency in ultrahigh altitude regions.

## Data Availability Statement

All datasets generated for this study are included in the article/supplementary material.

## Ethics Statement

The studies involving human participants were reviewed and approved by Ethics Committee of the Shengjing Hospital of China Medical University (Protocol Identification Number: 2017PS33K). Written informed consent from the participants' legal guardian/next of kin was not required to participate in this study in accordance with the national legislation and the institutional requirements.

## Informed Consent

The study involving human participants were reviewed and approved by Shengjing Hospital of China Medical University Ethics Committee (Shenyang, China). The study does not contain patients' personal information, fully protects the patients' privacy. The samples used in the study are all discarded samples after the clinical routine diagnosis and treatment, which has no impact on the routine diagnosis and treatment of patients. The patients do not conduct additional tests/checks due to participating in the study, and there is no harm to the patients, so we got through exemption from informed consent (Protocol Identification Number: 2017PS33K).

## Author Contributions

MY, XL, and JL: study conception and design. MY and XL: data acquisition. MY and JL: analysis and data interpretation. MY: drafting of the manuscript. JL: critical revision. All authors contributed to the article and approved the submitted version.

## Conflict of Interest

The authors declare that the research was conducted in the absence of any commercial or financial relationships that could be construed as a potential conflict of interest.
